# Within-host mathematical models to study antibody kinetics after the prophylactic Ebola vaccine in the Democratic Republic of the Congo

**DOI:** 10.1016/j.vaccine.2025.127707

**Published:** 2025-10-03

**Authors:** Irene Garcia-Fogeda, Steven Abrams, Stijn Vanhee, Maha Salloum, Benson Ogunjimi, Niel Hens

**Affiliations:** a Centre for Health Economics Research and Modelling Infectious Diseases (CHERMID), Vaccine & Infectious Diseases Institute (VAXINFECTIO), University of Antwerp, Antwerp, Belgium; b Global Health Institute (GHI), Family Medicine and Population Health (FAMPOP), University of Antwerp, Antwerp, Belgium; c Data Science Institute (DSI), Interuniversity Institute for Biostatistics and Statistical Bioinformatics (I-BioStat), Hasselt University, Hasselt, Belgium; d Department of Head and Skin, Ghent University, Belgium; e Antwerp Unit for Data Analysis and Computation in Immunology and Sequencing (AUDACIS), Antwerp, Belgium; f Antwerp Center for Translational Immunology and Virology (ACTIV), Vaccine & Infectious Diseases Institute (VAXINFECTIO), University of Antwerp, Antwerp, Belgium; g Department of Paediatrics, University Hospital Antwerp, Antwerp, Belgium

**Keywords:** Mechanistic models, Within-host mathematical models, Antibody kinetics, Ebola, Vaccination, Humoral immunity

## Abstract

Ebola virus disease remains a threat in different Sub-Saharan African countries more particularly in the Democratic Republic of Congo, where persistent outbreaks are driven by human populations living in close proximity to animal reservoirs. While vaccines like Ad26.ZEBOV and MVA-BN-Filo are safe and immunogenic, the dynamics of antibody responses after the two-dose regimen and booster vaccination are not fully understood. Within-host mathematical models offer valuable insights into disease dynamics and waning immunity, but data-driven mechanistic models of antibody kinetics remain scarce.

The present study seeks to elucidate the processes involved in antibody kinetics after the two-dose vaccine regimen with Ad26.ZEBOV and MVA-BN-Filo vaccines, followed by a booster dose vaccination with Ad26.ZEBOV, addressing challenges in inference for and implementation of within-host approaches.

By integrating established theoretical frameworks with recent empirical findings on antibody kinetics following Ebola vaccination, we illustrate how mechanistic modeling can enhance and refine our understanding of antibody dynamics. Specifically, we emphasize the distinction in the half-life of antibody responses at different vaccination time points and explore the role of vaccine antigens in eliciting an immunological response through the formation and activation of germinal center mediated response. Careful consideration was given to the development of a model that is both interpretable and practically feasible.

The half-life of the antibody response was found to be longer after booster vaccination compared to after the second vaccine dose, indicating a steadier decay process. This may be due to the improved quality of antibodies generated, the formation of memory B cells sustaining antibody production, and antigen-antibody binding.

This study highlights critical considerations for implementing within-host mechanistic models and the need for robust data to accurately estimate model parameters. Further research is essential to elucidate the decay dynamics of memory B cells and long-lived plasma cells, as these processes play a pivotal role in sustaining antibody-mediated immunity.

## 1 Introduction

Ebola virus disease is a severe and often fatal viral disease primarily transmitted through direct contact with bodily fluids of infected individuals or contaminated surfaces. The disease is characterized by symptoms such as fever, bleeding, and organ failure, with outbreaks occurring in Sub-Saharan Africa, mainly in parts of West Africa and the Democratic Republic of Congo (DRC), particularly in North Kivu, Ituri, and South Kivu [1]. Effective disease surveillance, training of specialized health care workers, and access to advanced therapeutics and vaccines are considered crucial factors to prevent larger Ebola outbreaks. Thus, ongoing efforts to implement an effective vaccination strategy in high-risk populations remain essential.

Currently, two licensed vaccines are recommended for the prevention of Ebola infection and severe disease. The first being a single-dose replication-competent viral vector vaccine manufactured as ERVEBO® by Merck, targeting the Zaire strain of the Ebola virus, and used in ring vaccination (i.e., vaccinating contacts of confirmed Ebola infected individuals) during ongoing Ebola outbreaks. A single dose of ERVEBO® has shown to be highly immunogenic, producing robust humoral responses [[2], [3], [4]]. Furthermore, the vaccine has shown to be highly efficacious and safe when used during recent outbreaks in Guinea and the DRC [5,6]. The second vaccine was jointly formulated by Janssen Vaccines & Prevention in collaboration with Bavarian Nordic, consisting of a two-dose heterologous vaccination regimen employing Ad26.ZEBOV (marketed as Zabdeno®) and MVA-BN-Filo (marketed as Mvabea®). The rationale of the two-dose vaccine regimen is to enhance and prolong both humoral and cellular responses. This prime-boost strategy has been shown to induce a robust and durable immune response in clinical studies with healthy individuals [[7], [8], [9], [10], [11]]. Ad26.ZEBOV is a monovalent vaccine designed to confer active, specific immunity against the Zaire Ebola virus. Meanwhile, MVA-BN-Filo is a polyvalent vaccine targeted to offer protection against a range of viruses, including the Sudan virus (SUDV), Ebola virus (EVD), Marburg virus (MARV), and the Tai Forest virus (TAFV). In addition, studies in children and people with HIV have shown that the booster dose with Ad26.ZEBOV has given an anamnestic immune response [12,13], suggesting its potential to enhance immunity in other populations. Nowadays, this vaccine regimen is recommended by the World Health Organization to be used during outbreaks for individuals at risk of Ebola exposure, and preventively, before outbreaks, for national and international first responders.

Although extensive research has been carried out on safety and immunogenicity of the two-dose vaccine regimen in different regions of West Africa and the DRC, less attention has been paid to studying the dynamics that are responsible for antibody production. Traditionally, antibody kinetics have been assessed using phenomenological models, which quantify trends without describing underlying biological processes. Alternatively, within-host mechanistic models could be used to describe the evolution in various biological populations, such as cells, thereby unravelling how specific components of the immune system respond and interact [14,15]. Typically, these dynamics are translated into a set of Ordinary Differential Equations (ODEs) with both population- and individual-level parameters governing changes in the biological processes that are described. A recent systematic review by Garcia-Fogeda et al. [14] found that many studies have relied upon phenomenological models rather than mechanistic ones. The latter can be further categorized into theory-driven and data-driven approaches. Theory-driven models, which were far more prevalent according to the systematic review, solely focused on the intrinsic properties of the model, exploring the impact of interventions on model outputs without validating them against observed data. In contrast, data-driven models use available data to inform and estimate model parameters and allow for the identification of the best-fitting model based on empirical data. By providing a deeper understanding of the biological mechanisms governing antibody kinetics, mechanistic models can help optimize vaccination strategies and improve immunological outcomes, advancing vaccine research beyond the descriptive capabilities of phenomenological models.

To date, several studies have explored within-host mechanistic models to study the antibody kinetics following the two-dose schedule with Ad26.ZEBOV and MVA-BN-Filo [13,[16], [17], [18]], focusing on short- and long- term humoral responses. However, challenges like computational time and identifiability of model parameters emerged largely due to limited immunological data and the design of data collection. While research on short- and long-lived plasma cells has progressed, the understanding of immune activation upon introduction of antigens and antibody dynamics post-booster dose remains limited.

This study focuses on the aforementioned two-dose vaccine regimen (Ad26.ZEBOV and MVA-BN-Filo) and booster dose with Ad26.ZEBOV, which was implemented in an open-label Phase II clinical trial (EBL2007, NCT04186000) in healthcare workers of Boende, DRC, which was part of the EBOVAC3 project [19]. Our primary aim is to generate new insights into the timing of vaccine-induced protection, evaluate the efficacy of prime-boost strategies, and quantify antibody decay. Particularly, this is the first study to integrate the booster dose with Ad26.ZEBOV into a within-host mechanistic modeling framework. Using immunogenicity data from the EBL2007 trial, we inform the mechanistic model parameters to not only describe antibody decay but also to elucidate the underlying biological mechanisms, such as B cell activation and memory cell formation. This mechanistic approach offers a level of biological interpretation that phenomenological models alone cannot provide, moving beyond descriptive patterns to a deeper understanding of immune dynamics.

The paper is organized as follows. In the Methods section the data and methodology employed to address the aforementioned objectives is introduced. Subsequently, the Results section details the application of mechanistic approaches to analyse the EBL2007 trial data. Finally, the Discussion section highlights the insights gained and reflects on the implications and lessons learned.

## 2 Methods

### 2.1 Data description

The participants in the study were healthcare providers and frontliners recruited from the Tshuapa province in the DRC [19], divided into two cohort groups based on two different booster schedules. Within the first year of the study, both cohorts were vaccinated with the same vaccine regimen, Ad26.ZEBOV (5 × 10^10^ viral particles [vp]) as a first dose and MVA-BN-Filo (1 × 10^8^ 50 % infectious units [Inf U]) as a second dose vaccination at a 56-day interval between vaccine administrations. Following the two-dose schedule, participants were randomized (1:1) to receive a booster dose of Ad26.ZEBOV either one (cohort 1) or two (cohort 2) years after the first dose. The reader is referred to Larivière et al. [19] for a comprehensive description of the study design.

The initial sample consisted of 700 participants, 676 meeting the per-protocol criteria, receiving both doses within the protocol-defined window, providing at least one post-vaccination immunogenicity sample, and having no major protocol deviations affecting the immune response [19]. Seroprevalence at baseline was low, with no reported prior EVD or EBOV vaccination or infection, and no evidence of outbreaks involving strains other than ZEBOV in the region, suggesting minimal pre-existing immunity or cross-reactivity [20,21].

**Fig. 1:**
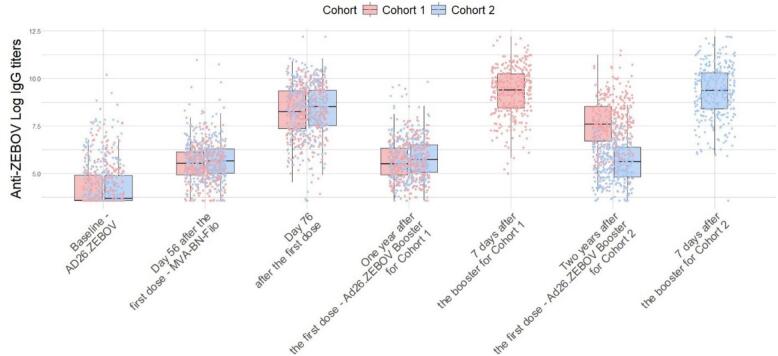
Boxplots of log-transformed antibody concentrations expressed in ELISA units/ml. At the time of the baseline measurement the first vaccine dose was administered, and at day 56 the second dose was administered. An additional antibody concentration measurement was performed 78 days after the first dose. The additional Ad26.ZEBOV dose, referred to as the prime-booster, was administered either one year (Y1) or two years (Y2) after the initial dose for cohort group 1 (in red) and cohort group 2 (blue), respectively. Subsequently, a blood sample was taken seven days after booster vaccination. The individual dots within the boxplots represent the variability of individual times within the visits at which the blood sample and vaccine administration were evaluated.

Among participants with available data on binding antibody responses around 21 days after the second dose with MVA-BN-Filo, 95.2 % met the responder threshold (>2.5× lower limit of quantification (LLOQ)). Anti-ZEBOV IgG titers that were below the LLOQ were considered left-censored (9 % in total, of which 53.2 % belong to the baseline measurement). Fig. 1 illustrates individual log-transformed antibody titers (ELISA units/ml) over time, encompassing the baseline measurement, vaccination moments and additional measurements based on samples collected for both cohort groups (i.e., cohort 1 shown in red, cohort 2 in blue).

The present study utilizes phenomenological models to quantify the association between antibody titers and vaccination status. This preliminary analysis aimed to assess whether the two cohorts exhibited similar immunological responses to the vaccination schedule, particularly to the booster dose, which was administered at different time points across cohorts. Identifying significant differences between cohorts would imply the need for cohort-specific parameters or structural adaptations in the subsequent mechanistic modeling to adequately reflect the underlying biological dynamics.

Subsequently, a linear mixed-effects model (LMM) was fitted, including both fixed effects for population-level and random effects for individual-specific variation and this model was fitted for each cohort group. Let *Y*_*ij*_ be the log antibody titer for individual *i* at measurement *j*, with *j* *=* *0* for pre-booster and *j* *=* *1* for post-booster. The variable Booster is equals to 1 as post-booster and 0 otherwise. This model allows us to assess the average effect of booster vaccination while accounting for individual-level heterogeneity in both antibody levels prior and posterior to the booster dose. The model is defined as follows:

(1) $$\begin{array}{c} Y_{\mathrm{ij}}=\left(\beta_{0}+b_{0i}\right)+\left(\beta_{1}+b_{1i}\right)*\mathrm{Booster}+\varepsilon_{\mathrm{ij}},\\ \varepsilon_{\mathrm{ij}}∼N\left(0,\sigma^{2}\right),b_{0i}∼N\left(0,\sigma_{0}^{2}\right),b_{1i}∼N\left(0,\sigma_{1}^{2}\right) \end{array}$$

The terms *b*_*0i*_ and *b*_*1i*_ denote individual-specific random intercepts applicable to two different measurement occasions, respectively. This model allows us to assess the average effect of booster vaccination while accounting for individual-level heterogeneity in both antibody levels prior and posterior to the booster dose.

Additionally, an LMM was used to quantify the impact of the booster dose within each cohort, accounting for the dependency between repeated antibody measurements (Appendix B). This model allowed estimation and testing of differences in mean titers between cohorts at each time point, as well as differences in booster effects between cohorts. The CIs were computed using the confint() function from the lme4 package in R, which uses the profile likelihood method. A significance level of 5 % and a *p*-value threshold of 0.05 were used for both analyses. These analyses were performed in R, version 4.2.3 [22].

While the linear mixed-effects model (LMM) was used to evaluate population-level changes in antibody titers and assess the comparability of cohort responses, it does not capture the underlying biological mechanisms that generate these dynamics. To address this, we applied a data-driven mechanistic modeling approach to gain deeper insight into the immunological processes involved in antibody production and decay following vaccination. Mechanistic models, described by ODEs (Eqs. (2), (3)), consist of compartments representing biological processes involved in antibody production. To account for left-censored observations, we employed a likelihood-based approach that integrates censored and non-censored data within a unified framework. Instead of imputing censored observations by using fixed values (e.g., LLOQ/2), a conditional likelihood formulation was used, ensuring that parameter estimation appropriately reflected the uncertainty introduced by censoring.

A variety of candidate models were considered in the process of identifying a biologically plausible yet parsimonious framework. These candidates were designed to reflect the key immunological mechanisms relevant to our study. While multiple structures were explored, the final model structure presented below was selected based on its alignment with the biological processes of interest and its ability to meet the selection criteria described at the end of this section.

### 2.2 Mechanistic model proposed by Nguyen et al. [23]

The proposed mechanistic model includes antigen (Ag), germinal center (GC) mediated response, and antibody (Ab) populations.

The antigen population represents the introduced (vaccine-)antigen, which is cleared by two processes: captured by antigen-presenting cells (APCs) and transported to the lymph nodes (rate δ_ag_), or binding with antigen-specific antibodies that form antigen-antibody complexes that are also deleted from the circulation (e.g. phagocytosed), with rate β_ag_.

The GC mediated response is subject to a delay process entailing a series of events to process an immunological signal (with delay governed by parameter τ_ag_). This delay is not intended to capture the full mechanistic detail of GC mediated response but rather to summarize, in a pooled form, the initiation of GC mediated response following antigen recognition. Once activated upon encountering an antigen induced in the Ag population, these events take place within the germinal center mediated response. Briefly, naïve B cells are activated by binding with antigens and engage with activated cognate T cells to initiate germinal center mediated response. This process leads to the migration of (mature) B cells into germinal centers, within the lymph nodes [24,25]. Afterwards, this process leads to B-cell proliferation, antibody affinity maturation, differentiation, proliferation, and consequently the production of antibodies, primarily through the generation of plasma cells. In this context, GC serves as a proxy for the integrated signal processing and functional output of GCs, particularly the activation of B cells and the subsequent differentiation that drives antibody production. Lastly, in the third equation, γ_ab_ is the antibody production rate in the germinal center mediated response, β_ab_ is the antibody decay upon binding with the antigen, and δ_ab_ represents the natural decay of antibodies.

(2) $$\begin{array}{c} \frac{\mathrm{dAg}}{\mathrm{dt}}=-\delta_{\mathrm{ag}}*\mathrm{Ag}-\beta_{\mathrm{ag}}*\mathrm{Ag}*\mathrm{Ab}\\ \frac{\mathrm{dGC}}{\mathrm{dt}}=\frac{\delta_{\mathrm{ag}}*\mathrm{Ag}-\mathrm{GC}}{\tau_{\mathrm{ag}}}\\ \frac{\mathrm{dAb}}{\mathrm{dt}}=\gamma_{\mathrm{ab}}*\mathrm{GC}-\beta_{\mathrm{ab}}*\mathrm{Ag}*\mathrm{Ab}-\delta_{\mathrm{ab}}*\mathrm{Ab} \end{array}$$

**Fig. 2:**
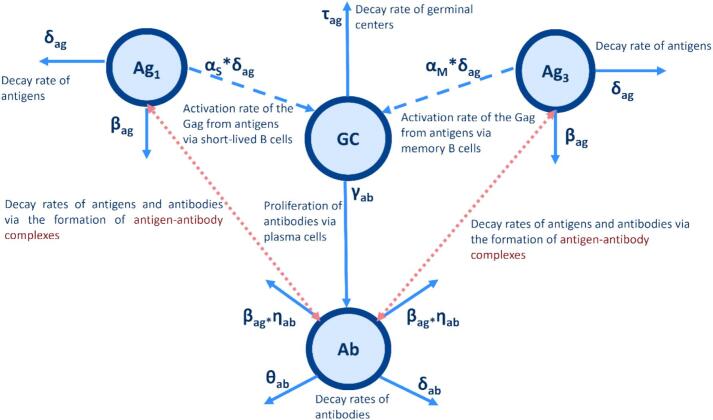
Schematic diagram of the mechanistic model structure corresponding to Eq. (3), showing the antigen introduction, germinal center mediated response, and antibody production dynamics.

The aforementioned model was restructured to better reflect our study scenario, in which a booster dose is administered, aiming for a more accurate representation of the underlying biological mechanisms. We found this structure to offer a biologically meaningful simplification that improved model identifiability and interpretability. Fig. 2 illustrates the schematic representation of the biological mechanisms incorporated into the restructured model (Eq. (3)), including antigen dynamics, germinal center mediated response, and antibody production following each vaccine dose and booster administration.

Building on the schematic representation shown in Fig. 2, the biological processes were mathematically formalized through a system of ODEs. The resulting model is defined as follows:

(3) $$\begin{array}{c} \frac{d{\mathrm{Ag}}_{1}}{\mathrm{dt}}=\left(-\delta_{\mathrm{ag}}*{\mathrm{Ag}}_{1}-\beta_{\mathrm{ag}}*{\mathrm{Ag}}_{1}*\mathrm{Ab}\right)*\left(1-I_{1}\left(t\right)\right)+\left(\emptyset_{1}\right)*I_{1}{\left(t\right)}_{u1-\varepsilon \leq t\leq u1+\varepsilon }\\ \frac{d{\mathrm{Ag}}_{3}}{\mathrm{dt}}=\left(-\delta_{\mathrm{ag}}*{\mathrm{Ag}}_{3}-\beta_{\mathrm{ag}}*{\mathrm{Ag}}_{3}*\mathrm{Ab}\right)*\left(1-I_{2}\left(t\right)\right)+\left(\emptyset_{2}\right)*I_{2}{\left(t\right)}_{u2-\varepsilon \leq t\leq u2+\varepsilon }\\ \frac{\mathrm{dGC}}{\mathrm{dt}}=\delta_{\mathrm{agS}}*{\mathrm{Ag}}_{1}+\delta_{\mathrm{agM}}*{\mathrm{Ag}}_{3}-\tau_{\mathrm{ag}}*\mathrm{GC}\\ \frac{\mathrm{dAb}}{\mathrm{dt}}=\left(-\delta_{\mathrm{ab}}*\mathrm{Ab}\right)*I_{3}{\left(t\right)}_{0<t\leq u2}+\gamma_{\mathrm{ab}}*\mathrm{GC}-\left(\theta_{\mathrm{ab}}*\mathrm{Ab}\right)*I_{4}{\left(t\right)}_{t>u2}-\beta_{\mathrm{abP}}*\left({\mathrm{Ag}}_{1}+{\mathrm{Ag}}_{3}\right)*\mathrm{Ab} \end{array}$$

$I_{k}\left(t\right)$ (k=1,2,3,4) represents an indicator function with value 1 when a specific condition applies. The measurement denoted by u_1_ and u_2_ indicate the individual times of second-dose and booster administration, respectively.

The first antigen is introduced via the Ad26.ZEBOV vaccine at baseline, which consists of 8.75 infectious units on the log10 scale [26], followed by MVA-BN-Filo vaccine administration on day 57 since the first dose, which contains an amount of 7.8 infectious units on the log10 scale [27]. The first antigen level is incorporated into the initial condition of Ag_1_, while the infectious units from the second dose are specified in $\emptyset_{1}$ using a Dirac delta function. In a similar manner, the population Ag_3_ describes the antigen of the booster dose, represented by $\emptyset_{2}$ implying a Dirac delta function.

The parameter τ_ag_ represents the decay of the germinal center mediated response, rather than 1/τ_ag_ as in Eq. (2). This adjustment is made because the activation of GCs following each vaccination moment is independent of their decay rate [28]. Limited antigen availability in the GCs after the first dose results in a response predominantly driven by naïve B cells that have matured and become activated upon encountering the antigen. After the second dose, memory B cells play a more prominent role in this process, with higher levels of antigen available due to the antibodies generated after the first dose that facilitate effective antigen presentation [28,29]. Specifically, the model incorporates two separate parameters, α_s_ and α_M_, which are proportional to the natural clearance rate of antigens (δ_ag_), representing the generation rates of short-lived plasma cells and memory B cells, respectively, from the GC mediated response. These are linked to antigen populations Ag_1_ (first and second dose with Ad26.ZEBOV and MVA-BN-Filo) and Ag_3_ (booster with Ad26.ZEBOV), respectively. While not explicitly included in the ODE system, α_S_ and α_m_ are incorporated through the generation rates of plasma cells as δ_agS_ = α_S_*δ_ag_ and δ_agM_ = α_M_*δ_ag_. From a biological point of view, early responses following primary vaccination (Ad26.ZEBOV and MVA-BN-Filo) are typically driven by the activation and differentiation of naïve B cells, producing mainly short-lived plasma cells, given that there was not pre-existing immunity and that the MVA-BN-Filo vaccine, confers immunity against additional serotypes. On the other hand, memory B cell responses are more dominant following re-exposure (booster), when pre-existing antibodies and B cells enhance antigen presentation and GC mediated response, facilitating the generation of memory B cells and long-lived plasma cells. While the model assumes a predominant contribution from short-lived plasma cells after primary vaccination and from memory B cells after the booster, we acknowledge that this representation may appear overly dichotomous. In reality, both cell types are likely generated following each dose, albeit in differing magnitudes and proportions. Due to the limited immunological data available, we chose to model these responses separately per dose to ensure parameter identifiability.

The parameter γ_ab_ reflects antibody production driven by plasma cells, irrespective of whether they originate from short-lived plasma cells or memory B cell. To evaluate antibody half-lives after the two-dose regimen and the booster, two decay parameters were estimated: δ_ab_ (from Y1 data) and θ_ab_ (from post-booster measurements). Initial values for δ_ab_ and θ_ab_ were informed by fitting separate LMM to their respective time interval, with time as a continuous covariate to log-transformed antibody concentrations. The slope coefficients from these models served an initial condition of the aforementioned decay rates. Additionally, the parameter β_abP_ is proportional, with proportionality factor η_ab_, to the decay of antigen after the formation of newly synthesized antibodies (β_ag_). The reason for this relationship is that the presence of antigens drives antibody production, so their clearance rate directly influences the rate at which antibodies are produced or maintained [30].

### 2.3 Mechanistic model implementation

Our first exclusion criteria to evaluate the models were poor Stochastic Approximation Expectation Maximization (SAEM) and poor convergence assessment. The goal for the algorithm is to first reach a neighbourhood around the maximum likelihood (ML) estimates during the initial phase and then progressively converge to the ML estimates for the model parameters. The second step, referred to as the convergence assessment, allows to execute a workflow of estimated parameters several times using different initial values as well as different seeds, enabling to assess the robustness of the convergence of the parameters. SAEM and convergence assessment were performed in Monolix, version 2023 [31].

Second, booster-related data as depicted in Fig. 1 (pre- and post-booster) were examined, and candidate models were fitted to the full dataset. Among models that met our selection criteria as described above, the best fit was determined based on Akaike's Information Criterion (AIC) [32], for more details see Supplementary Material Table C3. Pre-existing work on within-host mechanistic models, particularly those applied to Ebola and Hepatitis A [13,16,33], were of particular interest for evaluation in this study due to their incorporation of the dynamics of short- and long-lived antibody secreting cells. However, the mechanisms derived from these models were challenging to implement and infer given the available data, as it had fewer sampling points, it did not provide sufficient resolution to reliably estimate all model parameters of the aforementioned studied. Consequently, as they did not meet our inclusion criteria and required more extensive data to fully inform the model we adopted the model proposed by Nguyen et al. [23], presented below in Eq. (2).

Due to identifiability issues with the candidate model, parameter profiling and sensitivity analyses were conducted to refine the model, using individual parameter estimates from the SAEM algorithm near the ML neighbourhood. To explore parameter correlations and assess convergence robustness, we first ran five independent chains with varied initial values and seeds. This preliminary step allowed us to detect patterns of parameter dependence and convergence issues. Based on the insights from these runs, we performed a more extensive follow-up analysis using ten chains, systematically evaluating pairwise correlations to identify combinations contributing to poor convergence. As a result, these analyses enabled us to understand how changes in one parameter value influence others and to identify a plausible range of values for these, contributing to the development of a more parsimonious model.

A normal distribution with a proportional error was assumed for the raw antibody concentration data. To ensure positivity, the population-level parameters (fixed effects) are estimated on the log scale, and random effects are assumed to follow a normal distribution. As a result, the individual-specific parameters follow a lognormal distribution. The variability of these random effects assumes an inverse-Wishart distribution. Additional information on inference and parameter estimation can be found in Appendix A.

Finally, a non-parametric bootstrap approach using 250 bootstrap re-samples, was used to obtain confidence intervals for the estimated parameters of the final candidate model presented in the Results section. Sensitivity analyses and bootstrapping were conducted in R, version 4.2.3 [22]. Additionally, sensitivity analyses were performed using the full dataset that included both responders and non-responders, as well as a dataset excluding non-responders, and found no significant impact on the main findings. See Supplementary Table C5.

## 3 Results

Table 1 presents an overview of the demographic variables and summary measures related to antibody concentrations measured at the different visits. Although Mpox vaccination status and sex were not included as a covariate in our mathematical model, they are presented in the table due to its demonstrated relevance in immune responses during previous analyses of this trial [10]. Differences between sex within the cohorts, may have been amplified by the exclusion of pregnant and breastfeeding women, a common practice in clinical trials [19].

**Table 1:** Demographic variables and Anti-ZEBOV IgG measurements per healthcare worker (HCW) cohort and altogether. For continuous variables, mean and standard deviation (S.D.) are reported while for categorical variables, absolute frequencies (no.) and relative frequencies expressed as percentages (%) are shown. Y1 and Y2 refer to one year and two years after the initial vaccine dose, respectively.

Variable	Cohort 1 (*n* = 340)	Cohort 2 (*n* = 336)	All HCWs (*N* = 676)
Age, in years, mean (S.D.)	45.42 (11.51)	44.62 (12.46)	45.01 (11.99)

Sex, no. (%)			
Women	88 (25.88)	70 (20.83)	158 (23.37)
Men	252 (74.12)	266 (79.17)	518 (76.63)

Mpox vaccine, no. (%)			
Yes	59 (17.35)	67 (19.94)	126 (18.64)
No	281 (82.65)	269 (80.06)	550 (81.36)

Geometric mean antibody concentrations (in ELISA units/ml), (S.D.)			
Baseline	77.03 (4.74)	78.22 (5.08)	77.62 (3.44)
57 days post first dose	252.5 (13.44)	308.08 (18.09)	279.07 (10.99)
21 days post second dose	3825.67 (288.36)	4496.32 (341.47)	4144.98 (219.61)
Y1 after the first dose	279.7 (15.49)	339.06 (20.92)	307.82 (12.69)
7 days after the prime booster	10,781.58 (806.8)	**–**	10,781.58 (806.8)
Y2 after the first dose	1945.07 (159)	281.66 (17.86)	732.35 (47.44)
7 days after the prime booster	**–**	10,770.94 (873.08)	10,770.94 (873.08)

Following, we present the results of the phenomenological models used to assess the impact of booster vaccination. Next, we examine antibody kinetics, fitting the proposed mechanistic model in Eq. (3) and address the identifiability issues.

### 3.1 Phenomenological approach

Our objective with the phenomenological approach was to investigate whether the timing of prime-boost administration and the antibody response seven days post-booster exhibited comparable effects on antibody concentrations within each cohort. Table 2 reveals the estimates of the coefficients from the LMM fitted to the measurements prior and after the prime-booster dose per cohort. The overlapping confidence intervals, together with the results of two independent *t*-tests (see Table B1 in Appendix B), provide evidence of no statistically significant differences in mean antibody titers between the cohorts.

**Table 2:** Parameter estimates of the linear mixed effects model per cohort.

Parameter	Estimate (SE)	CI	P-value
**COHORT 1**			
Intercept	5.63 (0.06)	(5.51, 5.75)	< 0.01
7 days post-booster	3.64 (0.06)	(3.52, 3.77)	< 0.01

**COHORT 2**			
Intercept	5.64 (0.06)	(5.50, 5.77)	< 0.01
7 days post-booster	3.65 (0.07)	(3.50, 3.79)	< 0.01

### 3.2 Mechanistic approach

We now turn to biological understanding of antibody kinetics. First, the abovementioned model was fitted to the full dataset, using the parameter estimates obtained from fitting the model to the Y1 data as initial values. Proportionality factors were set to positive values, assuming that α_S_ and α_M_ were equal, and that η_ab_ was equal to 1. On the other hand, initial decay rates of 0.009 and 0.004 were based on estimates from the LMM fitted to the Y1 data and the booster process. After running the model and applying the selection criteria defined in the Methods section, we examined bivariate plots to evaluate how changes in one parameter influenced values for others. The most divergent parameter was identified and fixed to the arithmetic mean of its conditional values across chains. This iterative process was repeated until the convergence criteria were met. As a result, Table 5 presents the most parsimonious model from Eq. (3), pooling all parameter estimates from the different chains (i.e., 10 in total), which are initialized using different starting values and seeds. Further details on the convergence assessment and sensitivity analyses can be found in Appendix D, with parameter definitions summarized in Table C4 of Appendix C.

**Table 5:** Parameter estimates using the most parsimonious model from Eq. (3) for cohorts 1 and 2, fixing the parameters {α_s_ = 0.4, δ_ag_ = 0.4, η_ab_ = 3.88, τ_ag_ = 0.24}, and assuming a normal distribution and a proportional error model for the raw antibody titers. The fixed effects are on the log-scale and the random effects follow a normal distribution. The standard error estimates (S.E.) for the estimators of the model parameters include within- and between-chain variability based on 10 chains that were initialized with different starting values. The standard error estimates for the transformed parameters are derived using the Delta Method. The bootstrap-based 95 % percentile confidence intervals (CIs) are calculated based on a non-parametric bootstrap approach with 250 bootstrap samples included.

Parameter	Definition	Parameter estimate (S.E.)	Transformed parameters (S.E.)	95 % CI
β_ag_	Decay of antigens when are combined with newly synthesized antibodies forming so-called antigen-antibody complexes	0.0413 (0.0015)	0.0700 (0.0039)	(0.06393, 0.0756)
α_M_	Proportional rate of memory B-cells	3.1243 (0.2285)	7.7069 (0.7007)	(5.9434, 8.8697)
γ_ab_	Proliferation of antibodies induced by plasma cells	352.1254 (16.3857)	1041.5965 (60.2009)	(968.4236, 1172.3990)
δ_ab_	Natural decay of antibodies after the two-dose vaccine regimen	0.0097 (0.0001)	0.0112 (0.0002)	(0.0108, 0.01150)
Ab(0)	Baseline antibody titers	42.3501 (3.7263)	283.2544 (34.0430)	(234.8518, 322.0862)
θ_ab_	Natural decay of antibodies after the booster dose	0.0057 (0.0001)	0.0074 (0.0002)	(0.0068, 0.0078)
ɛ_βag_	Random effects of β_ag_	0.5278 (0.0417) 0.9028 (0.0539) 1.0845 (0.0342) 0.1449 (0.0144) 1.900 (0.0818) 0.2584 (0.0227)		
ɛ_αM_	Random effects of α_M_			
ɛ_γab_	Random effects of γ_ab_			
ɛ_δab_	Random effects of δ_ab_			
ɛ_Ab(0)_	Random effects of Ab(0)			
ɛ_θab_	Random effects of θ_ab_			

Antigen clearance occurs through two processes with decay rates δ_ag_ and β_ag_. The first indicates the rate of antigen uptake by APCs and has a fixed value of 0.4, suggesting a half-life of 2 days. This value provides additional insight into the timing of antigen decay, which is consistent with literature stating that antigen clearance typically occurs within the first week post-vaccination [34,35]. The parameter β_ag_ reflects the effectiveness of the immune system in clearing antigens through the formation of antibody-antigen complexes. With a value of 0.07 (95 % CI: 0.063, 0.075), this indicates that antigen clearance via antibody binding is relatively slow. This could be due to the lack of pre-existing antibodies prior to vaccination, or from the fact that most antigens are taken up by APCs.

The results displayed by the GC mediated response indicate that it takes approximately 4.17 days to create an immunological signal, consistent with prior findings [24,25]. Additionally, the proportional rates from the short-lived plasma cells and memory B cells (α_S_, α_M_) suggest that memory B cells are generated more rapidly, as their rate of response is higher than that of the short-lived plasma cells (7.71 vs 0.4) [28,36]. This is consistent with the longer lifespan of memory B cells compared to short-lived plasma cells, which are cleared after days following vaccination [13].

Turning to the antibody population, γ_ab_ represents a rate parameter that quantifies the antibody production. This rate is determined by the number of plasma cells actively producing antibodies, which is estimated to be approximately 1041 plasma cells. Once antibodies bind to antigens, they are no longer available to neutralize additional antigens, and they are cleared in the process of an antigen-antibody interaction (β_abP_) with a rate of 0.27 (i.e., β_ag⁎_η_ab_) with 95 %CI: (0.017, 0.020). The proportionality factor η_ab_ of the aforementioned parameter exceeds one, indicating effective antigen clearance. Since the antigens peak at two days post vaccination and decay rapidly, making antibody clearance via binding (β_abP_) significant only when these antigens are still present after vaccination. Given this and the variability in data intervals informing antibody decay rates, we estimate half-lives relying on an exponential decay process rather than focusing on the mechanistic model's analytical solution for the antibody titers. The parameter δ_ab_ represents the natural decay of the antibodies after the two-dose vaccine regimen (Ad26.ZEBOV, and MVA-BN-Filo 57 days later), with a half-life of 2 months (62 days with 95 % CI: (57.76, 69.3) days). Meanwhile, the parameter θ_ab_ reflects the antibody decay after the prime-booster dose (Ad26.ZEBOV), resulting in a half-life of approximately 3 months (94 days with 95 % CI: (86.64, 105.02) days), indicating a higher persistence of antibodies following the booster dose with Ad26.ZEBOV. The parameter Ab(0), estimated at 283.25 EU/mL, reflects the typical individual's baseline antibody level before vaccination, accounting for both left-censored observations and inter-individual variability. This value is derived from a population-level fit and may be higher than the empirical GMC due to model-based integration of uncertainty and data from subsequent timepoints.

**Fig. 3:**
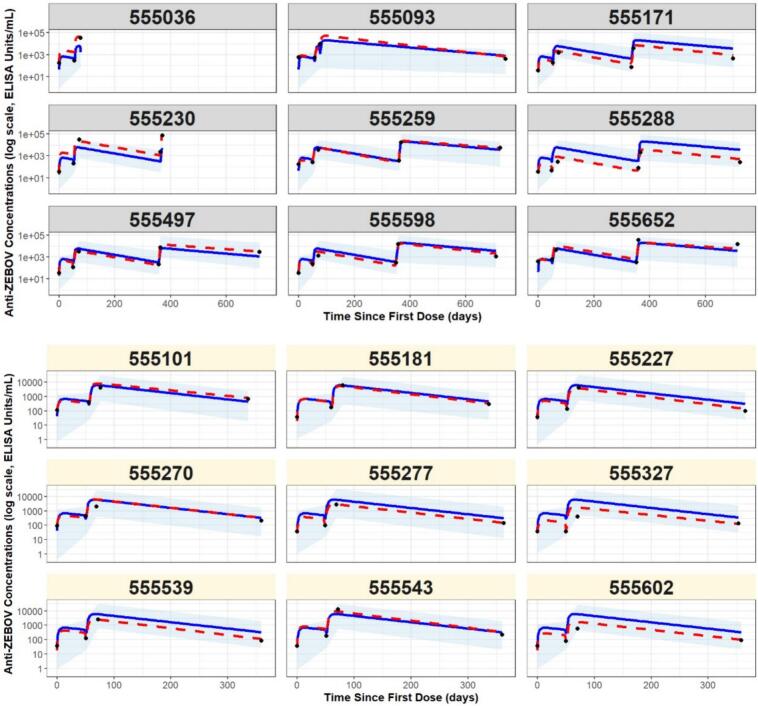
Predicted log-transformed IgG titer profiles given by the estimated individual-level model, which is the parsimonious model from the Eq. (3) with fixed parameters {α = 0.4, δ_ag_ = 0.4, η_ab_ = 3.88, τ_ag_ = 0.24}.}. In the upper panel cohort 1 is represented in the grey boxes, and in the lower panel the cohort 2 is represented in light yellow boxes. The black dots represent the observed data, the red dashed curves are the approximated conditional mean curves from SAEM, and the blue curves depict the population-level fits based on the estimated fixed effects and individual-specific design characteristics, without incorporating individual-specific random effect estimates. The blue shaded areas represent the 2.5 % and 97.5 % predicted percentiles, which account for inter-individual variability by including random effects sampled from the estimated population-level distribution, rather than being based on individual-specific estimates.

Fig. 3 displays the observed data for each participant, as well as the predicted profiles given by the estimated individual-level model. Moreover, it can be observed that the predicted values correspond well with the observed data. Based on this graphical depiction, the model does not show a lack-of-fit to the available data. Since no substantial differences between cohorts were found in the phenomenological analysis, we assumed shared parameter values across cohorts in the mechanistic model. Due to identifiability and convergence issues, assessing cohort-specific mechanisms was not feasible and remains an open question for future study. Further model diagnostics are presented in Appendix D.

## 4 Discussion

The present study was designed to analyse the antibody dynamics using within-host mechanistic models. The first question sought to determine the antibody half-lives after the second dose and the subsequent booster dose. The estimated half-lives are crucial for understanding the waning processes and the persistence of immune responses against infection [37,38]. Our findings indicate antibody half-lives of two months (i.e., 62 days, 95 % CI: (57.76, 69.3) days) after the two-dose vaccine regimen and approximately three months (i.e., 94 days, 95 % CI: (86.64, 105.02) days) after booster vaccination. In contrast, previous studies, such as those by Pasin et al. [13] and Alexandre et al. [16], estimated shorter antibody half-lives of around 24 days (95 % CI: 22, 26) after the second dose, while others found a more prolonged persistence [9]. Studies using ERVEBO vaccine studies reported a half-life of 28 days [4]. These differences as compared to our estimates can be explained in part by the inclusion of three different decay rates for the antibody population in our model, which allowed us to disentangle different processes contributing to the overall decrease of antibody levels. Antibody decay should be viewed in terms of overall levels over time, considering multiple overlapping processes. In this study, the antibody measurements were specific to the Zaire ebolavirus (ZEBOV) glycoprotein, as captured by the FANG ELISA assay. While this assay may pick up minimal background signal, significant cross-reactivity with other filovirus strains is unlikely and could not be directly modeled due to lack of specific antigenic data [28,39].

In particular, the mechanistic model allowed us to separately estimate decay rates linked to waning of antibody responses by physiological processes, antigen-driven clearance, and antibody production in response to booster-induced antigen presentation. This multi-process view provides a more nuanced understanding than models assuming a single decay rate. Our findings suggest that antibodies persist longer after the booster dose, thereby extending humoral immunity.

While this study focused on the half-lives of binding antibodies, it is also important to understand how antibody levels persist after they begin to decay. Our findings on antibody persistence, derived from the decay rates in the mechanistic model, indicate that by day 100 post first-dose, antibody levels are expected to decline with respect to the 2.5-fold increase above the LLOQ serving as a reference for the immunogenicity threshold. Following the booster dose, the decay is steadier, in particular it starts to decline towards the immunogenicity threshold at day 307. Individual variability in immune responses suggests that not all individuals will reach this threshold at the same time, with many maintaining antibody levels above this value.

Consistent with literature, binding antibody concentrations were persisting for more than 3 years [11,[40], [41], [42], [43]], although at lower levels than some studies have reported [46]. Choi et al. [44] observed antibody persistence lasting 4 to 5 years after the second dose and noted a 54-fold increase 7 days post-booster, whereas our study recorded a 44.75-fold increase 95 % CI: (35.38, 56.60)- and 34.79-fold increase 95 % CI: (22.79, 53.11) per cohort at the same interval. Similarly, Manno et al. [45] reported persistence beyond 3 years and found a 101- and 44-fold increase in antibody levels 21 and 7 days post-booster, respectively. These findings highlight the importance of carefully timed sampling to accurately assess long-term antibody titers. Comparing trials based on isolated cross-sectional data may therefore lead to misleading conclusions regarding the duration of antibody positivity, as there is no established correlate of protection (CoP). To accurately evaluate long-term antibody persistence, studies must account for the full trajectory of antibody decay.

Importantly, the phenomenological analysis revealed no substantial differences in antibody responses between cohorts, suggesting that the timing of booster dose administration could be relatively flexible. This has valuable implications for immunization strategies in remote or resource-limited areas [46]. The absence of observed differences in the overall antibody kinetics implies that the underlying parameters of the mechanistic model, such as antigen clearance, memory B cell response, or decay processes, may be similar between cohorts. However, this assumption remains to be verified. Due to data limitations and identifiability issues, our model did not allow us to robustly investigate whether such mechanistic processes were different across cohorts. Future studies should study this effect as a covariate, to determine whether these underlying immune mechanisms are truly shared.

Neutralizing antibodies are essential as they can neutralize antigens or viruses. While our study does not directly measure neutralizing antibodies, the parameter β_abP_ in our model, with a proportionality factor of 3.88, suggests significant antibody-antigen binding, potentially indicating neutralization. To contextualize our findings, prior studies have demonstrated the persistence and variability of neutralizing antibodies following vaccination. Choi et al. [47] reported the presence of Anti-Ad26 neutralizing antibodies in small infants, while Bockstal et al. [40] observed robust neutralizing responses against Ebola virus, specifically the Zaire strain (ZEBOV) but lower rates for SUDV and MARV. Mutua et al. [11] further found that neutralizing antibodies persisted up to day 360 following a two-dose regimen. This highlights the importance of future studies to determine whether the antigen-binding interactions identified in our model also translate into neutralizing functionality, which is critical for protective immunity.

These findings may be limited by the focus solely on binding antibodies, without fully capturing short- and long-term humoral responses. Short-lived antibody secreting cells provide an immediate response, while long-lived antibody secreting cells ensure sustained antibody production [48]. Recent studies suggest that short-term antibodies may persist for up to a year post-prime immunization [13,16], while long-term responses show a greater durability between 5 and 15 years [13,16]. While our model distinguishes between short-lived plasma cells and memory B cells in germinal center mediated response, additional follow-up data would strengthen these distinctions. Nonetheless, defining additional components of immunity further increases the need for sufficient data to infer all relevant processes [23]. Rather than just estimating antibody decay, we inferred meaningful biological quantities, such as antigen-antibody binding rates, and the relative generation of memory vs. short-lived plasma cells in GCs. This is in particular reflected in the parameters α_M_ being much higher than α_S_, suggesting a robust memory B cell response following the booster dose, consistent with expected immunological mechanisms [49] [50]. On the other hand, including antigen decay dynamics (δ_ag_, β_ag_) and their interaction with antibody levels provides insight into how long antigen is present to stimulate the immune system.

We recommend starting with simpler models before progressing to more complex ones, particularly given the challenges of computational time and parameter identifiability. While there is extensive literature on solving mechanistic approaches with standard ODE solvers (e.g., in MATLAB or R), software that integrate ODE solving with parameter estimation including random effects (e.g., mixed effects modeling) is less available. In these cases, software like Monolix (frequentist/Bayesian) [31]and JAGS (Bayesian) [14], as well as Stan, which uses MCMC with the no-U-turn sampler (NUTS) [51], are available. The choice between these tools often depends on the statistical approach, computational efficiency, flexibility in solving differential equations, user interface, and licensing constraints. Ultimately, model development should strive for parsimony while also considering practical feasibility, especially when working with limited data.

An important limitation of this study is the scarcity of immunological and longitudinal data, which constrains model accuracy. Direct information on the processes driving antibody dynamics was unavailable, leaving binding antibody levels as the only source of information. Parameter estimation relied on backward fitting, requiring fixed parameters to address identifiability issues, which is a common challenge that often leads to simplified models [13,17]. By contrast, studies with richer longitudinal data have successfully identified key biological parameters [33,52,53]. Further research should explore the data needed to fully inform these models. Despite these limitations, this study provides valuable insights into antibody kinetics and immune processes.

We did not assess the effect of covariates on our model parameters due to identifiability issues and the lack of sufficient data to support these analyses. Incorporating covariates would require estimating additional parameters, adding complexity to a model where parameter estimation was already challenging. However, previous studies have highlighted that factors such as sex, vaccination against Mpox, and geographic region have an influence on antibody kinetics [4,10,13,16,47]. While covariate effects have been partly studied using simpler models [10], it remains to be determined whether the parameters driving antibody production, such as those estimated in our mechanistic framework, differ meaningfully between cohorts. Including such an analysis in our study would have largely replicated existing work, without providing further biological insight into the mechanistic processes specifically involved in the booster antibody response.

This study did not assess the role of B cell subpopulations, specifically memory B cells (MBCs) nor long-lived plasma cells (LLPCs), but how the germinal center mediated response gets activated after each dose producing plasma cells which produce binding antibodies. This is important because within the GCs, some B cells differentiate into LLPCs, which reside in the bone marrow and produce high-affinity antibodies, ensuring long-term protection. Others become MBCs, which persist in circulation and can quickly differentiate into antibody-secreting cells upon re-exposure to the antigen. Unlike LLPCs, which maintain antibody production regardless of antigen presence, MBCs can contribute to a broader immune response and can recognize antigen variants. [25,26]. Recent work by Xu et al. [54] has further highlighted the complexity of these processes, demonstrating that antibody repertoire reshaping and memory dynamics are influenced by sequential or heterologous exposures to different viral infections. While our model distinguishes between short-lived plasma cells and memory B cells indirectly, it does so through a simplified compartment representing the GC mediated response, abstracting away the full complexity of the cellular processes within GC mediated response. This modeling choice was deliberate, balancing biological plausibility with identifiability and data limitations. Detailed modeling of B cell activation, T cell help, and affinity maturation would require extensive immunological measurements not available in the current dataset. Instead, we tracked antigen and antibody concentrations, two components for which direct data were available, using a structure that preserves the essential Ag → GC → Ab biological processes. In future investigations, both MBCs and LLPCs could be added as compartments, to better understand the longevity of immunity conferred by this vaccine regimen.

## 5 Conclusion

This study set out to assess the feasibility of implementing within-host mechanistic approaches to gain a deeper understanding of antibody dynamics. Our findings reveal that antibody half-lives after the booster dose is greater than after the second dose. Additionally, we quantified key biological processes underlying antibody dynamics, contributing to a more comprehensive understanding of these mechanisms.

The challenges addressed in inferring these models have provided valuable insights into antibody responses and lay the groundwork for future research on binding antibodies and memory B-cell decay. Further research should refine these models and identify necessary data.

In conclusion, these findings, together with future investigations, can be used to develop targeted interventions aimed at when to implement vaccination schedules. While a definitive CoP for Ebola vaccines has not yet been established, estimating when antibody levels fall below certain thresholds may still offer valuable guidance for booster timing. Importantly, our mechanistic model captures between-individual variation in immune responses, which can inform tailored guidance on vaccine schedules among healthcare workers in this case. This is especially relevant in resource-limited areas, where identifying subgroups at risk of earlier waning immunity may be critical for preventing future outbreaks.

## CRediT authorship contribution statement

**Irene Garcia-Fogeda:** Writing – review & editing, Writing – original draft, Visualization, Software, Resources, Methodology, Investigation, Formal analysis, Data curation, Conceptualization. **Steven Abrams:** Validation, Supervision, Methodology, Investigation, Formal analysis, Conceptualization. **Stijn Vanhee:** Validation, Resources, Methodology, Formal analysis, Conceptualization. **Maha Salloum:** Validation, Resources, Investigation, Conceptualization. **Benson Ogunjimi:** Validation, Methodology, Funding acquisition, Formal analysis, Conceptualization. **Niel Hens:** Validation, Supervision, Project administration, Methodology, Investigation, Funding acquisition, Formal analysis, Conceptualization.

## Funding

The EBOVAC3 project has received funding from the IMI2 Joint Undertaking under grant agreement No 800176 (IMI-EU). This Joint Undertaking receives support from the 10.13039/100010661European Union's Horizon 2020 research and innovation programme, 10.13039/100013322European Federation of Pharmaceutical Industries and Associations (EFPIA) and the Coalition for Epidemic Preparedness Innovations (CEPI). This project has received funding from the 10.13039/501100000781European Research Council (ERC) under the European Union's Horizon 2020 research and innovation programme (Grant agreement No. 851752) and the European Union's Horizon 2020 research and innovation programme grant agreement 851752-CELLULO-EPI (B.O.)

## Declaration of competing interest

The authors declare the following financial interests/personal relationships which may be considered as potential competing interests: Irene Garcia-Fogeda reports financial support was provided by University of Antwerp Faculty of Medicine and Health Sciences. Niel Hens (NH) reports that the Universities of Antwerp and Hasselt have received funding for advisory boards and research projects of MSD, GSK, JnJ, Pfizer outside the proposed work If there are other authors, they declare that they have no known competing financial interests or personal relationships that could have appeared to influence the work reported in this paper.

## Data Availability

The data that has been used is confidential.

## Appendix A Supplementary data

Supplementary material Image 1
