# Supplementary material for: Within-host mathematical models to study antibody kinetics after the prophylactic Ebola vaccine in the Democratic Republic of the Congo
Source: Vaccine. 2025 Oct 3;64:None. doi: 10.1016/j.vaccine.2025.127707 (PMC12519101; doi:10.1016/j.vaccine.2025.127707)
Supplement: Supplementary file 1 — Supplementary material [file mmc1.docx]

**Within-host mathematical models to study antibody kinetics after the prophylactic Ebola vaccine in the Democratic Republic of the Congo**

Irene Garcia-Fogeda^1*^, Steven Abrams^2,3^, Stijn Vanhee^4^, Maha Salloum^2^, Benson Ogunjimi^1,5,6,7^, Niel Hens^1,3^

^1^ Centre for Health Economics Research and Modelling Infectious Diseases (CHERMID), Vaccine & Infectious Diseases Institute (VAXINFECTIO), University of Antwerp, Antwerp, Belgium.

^2^ Global Health Institute (GHI), Family Medicine and Population Health (FAMPOP), University of Antwerp, Antwerp, Belgium

^3^ Data Science Institute (DSI), Interuniversity Institute for Biostatistics and statistical Bioinformatics (I-BioStat), Hasselt University, Hasselt, Belgium

^4^ Department of Head and Skin, Ghent University, Belgium

^5^ Antwerp Unit for Data Analysis and Computation in Immunology and Sequencing (AUDACIS), Antwerp, Belgium

^6^ Antwerp Center for Translational Immunology and Virology (ACTIV), Vaccine & Infectious Diseases Institute (VAXINFECTIO), University of Antwerp, Antwerp, Belgium

^7^ Department of Paediatrics, University Hospital Antwerp, Antwerp, Belgium

*Corresponding author: [irene.garcia-fogeda@uantwerpen.be](mailto:irene.garcia-fogeda@uantwerpen.be)

***Appendices***

***Appendix A. Parameter estimation***

The method of estimation combines Maximum Likelihood Estimation (MLE) and the SAEM algorithm. Following, samples from the conditional distribution are used to compute the conditional means and conditional standard deviations of the individual parameters, using the Metropolis-Hastings algorithm.

Parameters are estimated by non-linear mixed-effects models. Monolix uses Gaussian distributions that allow some transformations of the parameters of interest to be normally distributed. In particular, these models integrate a population parameter (ψ_pop_) and a random effect denoted by Ω_i_, which is log-normally distributed with E[Ω_i_] = 1.In the Eqs below, (ψ_i_ ) stands for the fixed effect and the median of the distribution, while ω defines the standard deviation of the random effects, which is interpreted as the inter-individual variability.

$$\log\left( \psi_{i} \right)\sim\boldsymbol{N}\left( \log\left( \bar{\psi_{i}} \right),\omega^{2} \right)\approx\log\left( \psi_{i} \right)=\log\left( \bar{\psi_{i}} \right)+\Omega_{i}\approx\psi_{i}=\bar{\psi_{i}}e^{\Omega_{i}}$$

Ωi ~ N(0, ω^2^)

*Eqs.(1)*

In addition, a constant error model was chosen for the likelihood. Being Y_ij_ the outcome, in our case the antibody concentrations, we can define the likelihood as follows:

$$U(Y_{ij}) = U(f(t_{ij}, \psi_{i})) + g(U(t_{ij}, \psi_{i}), x_{i})*\sigma_{ij}^{2}$$

*Eq (2)*

Where ψ_i_ is the parameter vector of the structural model, which integrates the population parameter with the i individual random effects. The function g(x) defined the residual error and depends of some additional parameters xi and is being multiplied by standardized Gaussian random variables ~N(0, 1). Thus, f(t_ij_, ψ_i_) and g(t_ij_, ψ_i_, x_i_) are the conditional mean and standard deviation of Y_ij_, respectively. Working with a proportional error model for the likelihood would mean in this case that g(x) = b*f(x). Moreover, a normal transformation of y was used in the formula defined above (i.e., U(y) = y).

***Appendix B. Phenomenological model***

**Table B1. Independent two-sample t-test of differences in mean log-transformed antibody titer concentration 7 days post prime-booster between the two cohorts cohort (µ_a_ and µ_b_, respectively for cohort 1 and 2) and differences in mean difference in log-transformed antibody titer concentration between the measurements 7 days after the prime-boost and prior to the booster dose (the latter denoted by µ_c_ for cohort 1 and µ_d_ for cohort 2).**

| Test | P-value |
| --- | --- |
| H_0_: µ_a_ - µ_b_ = 0 | 0.26 |
| H_0_: (µ_a_ - µ_c_) – (µ_b_ - µ_d_) = 0 | 0.19 |

Table B2 provides all measurements and interaction effects between cohorts in the linear mixed-effects model (LMM). It is noteworthy that differences between cohorts in the second year are anticipated, given that cohort 1 received the booster dose a year earlier. Consequently, based on this analysis, we can infer that there is no evidence on supporting distinctions between the cohorts. The Equations for this analysis are defined as follows:

$$Y_{ij}= \beta_{0i}+\beta_{1j}*{Measurement}_{j}+ \beta_{2}*Cohort+\beta_{3j}*Cohort*{Measurement}_{j}+\varepsilon_{ij}$$

*ɛ_ijk_ ~ N(0, σ^2^)*

*β_0i_ = β_0_ + b_i0_, where b_i0_ ~ N(0*, $\sigma_{0}^{2}$*)*

*Eqs (3)*

**Table B2. Parameter estimates of the linear mixed effects including all the measurements and all the cohorts.**

| **Parameter** | **Estimate (SE)** | **CI** | **P-value** |
| --- | --- | --- | --- |
| Intercept | 4.35 (3.35) | (4.22, 4.47) | 0.19 |
| MVA-BN-Filo: Day 57 | 1.18 (4.74) | (1.05, 1.31) | 0.8 |
| Day 78 | 3.89 (4.74) | (3.76, 4.02) | 0.41 |
| Year 1: Ad26.ZEBOV booster cohort 1 | 1.28 (4.74) | (1.15, 1.41) | 0.78 |
| 7 days after the prime-booster: cohort 1 | 4.92 (4.74) | (4.79, 5.06) | 0.29 |
| Year 2: Ad26.ZEBOV booster cohort 2 | 3.22 (4.74) | (3.08, 3.35) | 0.49 |
| 7 days after the prime-booster: cohort 2 | 4.92 (4.74) | (4.79, 5.06) | 0.29 |
| Cohort 2 | 0.01 (0.09) | (-0.16, 0.19) | 0.89 |
| Cohort 2* MVA-BN-Filo: Day 57 | 0.18 (0.09) | (0.00, 0.37) | 0.04 |
| Cohort 2*Day 78 | 0.15 (0.09) | (-0.03, 0.33) | 0.11 |
| Cohort 2* Year 1: Ad26.ZEBOV booster cohort 1 | 0.17 (0.09) | (-0.01, 0.36) | 0.06 |
| Cohort 2* Year 2: Ad26.ZEBOV booster cohort 2 | -1.94 (0.09) | (-2.13, -1.76) | <0.01 |

***Section C. Mechanistic approach***

**Table C3: Candidate mathematical models that complied with the selection criteria.**

| **Model** | **Description** | **Key Features** | **Reasons for Exclusion** | **AIC Y1** | **AIC Full Dataset** |
| --- | --- | --- | --- | --- | --- |
| Andraud et al [1] | Mechanistic model of antibody response post-vaccination | Short-lived and long-lived antibody secreting cells, and antibodies | Poor fit in the observed vs. predicted diagnostics, overestimation of the booster response in the prediction percentiles, and parameter convergence issues affecting both fixed and random effects | 54691.64 | 40097.75 |
| Nguyen et al [2] | Mechanistic model to assess the immune response of vaccinated monkeys exposed later on to Ebola | Antigen, Germinal center-mediated immune response, antibodies | This model provided the best fit, showed no diagnostic issues, and achieved better parameter convergence compared to alternative models. | 44487.21 | 35212.33 |
| Le et al [3] | Phenomenological model that uses a piecewise model describing antibody kinetics using an exponential rise until a peak time, followed by exponential decay | Biphasic antibody response, taking into account exposure effect | Poor fit in the observed vs. predicted diagnostics, overestimation of the booster response in the prediction percentiles, and parameter convergence issues affecting both fixed and random effects | 53569.31 | 40513.27 |
| Farrington et al [4] | Phenomenological model describing the time-dependent antibody concentrations after vaccination | Captures an antibody response with an initial rise followed by exponential decay | Poor fit in the observed vs. predicted diagnostics, overestimation of the booster response in the prediction percentiles, and parameter convergence issues affecting both fixed and random effects | 54680.10 | 39426.06 |

**Table C4: Glossary of the parameters presented in Eqs 3.**

| **Parameter** | **Definition** |
| --- | --- |
| δ_ag_ | Natural decay of antigens that are captured by antigen-presenting cells such as macrophages and transported to the lymph nodes |
| β_ag_ | Decay of antigens when are combined with newly synthesized antibodies forming so-called antigen-antibody complexes |
| ϕ_1_ | Dirac Delta function representing the infectious units of the MVA.BN-Filo vaccine administered 57 days post first dose |
| ϕ_2_ | Dirac Delta function representing the infectious units of the Ad26.ZEBOV vaccine administered a year after the first dose |
| α_S_ | Proportional rate of the short-living cells |
| δ_agS_ | Rate at which germinal centers are activated due to the short-living cells (α_S_) and the natural decay of antigens (δ_ag_) |
| α_M_ | Proportional rate of memory B-cells |
| δ_agM_ | Rate at which germinal centers are activated due to the memory B-cells (α_M_) and the natural decay of antigens (δ_ag_) |
| τ_ag_ | Natural decay of germinal centers |
| δ_ab_ | Natural decay of antibodies after the two-dose vaccine regimen |
| γ_ab_ | Proliferation of antibodies induced by plasma cells |
| θ_ab_ | Natural decay of antibodies after the booster dose |
| η_ab_ | Proportion of binding antibodies that attached to a single antigen |
| β_abP_ | Rate of antibodies that die out after binding with the antigen that is dependent on η_ab_ and β_ag_ |

**Table C5: Results of responders to the vaccine. Parameter estimates using the most parsimonious model from Eqs. (3) for cohorts 1 and 2, fixing the parameters {α_s_ = 0.4, δ_ag_ = 0.4, η_ab_ = 3.88, τ_ag_ = 0.24}, and assuming a normal distribution and a proportional error model for the raw antibody titers. The fixed effects are on the log-scale and the random effects follow a normal distribution. The standard error estimates (S.E.) for the estimators of the model parameters include within- and between-chain variability based on 10 chains that were initialized with different starting values. The standard error estimates for the transformed parameters are derived using the Delta Method.**

| **Parameter** | **Definition** | **Parameter estimate (S.E.)** | **Transformed parameters (S.E.)** |
| --- | --- | --- | --- |
| β_ag_ | Decay of antigens when are combined with newly synthesized antibodies forming so-called antigen-antibody complexes | 0.0521 (0.0022) | 0.0965 (0.0005) |
| α_M_ | Proportional rate of memory B-cells | 0.9710 (0.0493) | 1.6213 (0.1284) |
| γ_ab_ | Proliferation of antibodies induced by plasma cells | 587.4326 (25.3151) | 1355.9550 (72.7825) |
| δ_ab_ | Natural decay of antibodies after the two-dose vaccine regimen | 0.0101 (0.0001) | 0.0120 (0.0001) |
| Ab(0) | Baseline antibody titers | 51.1863 (4.9687) | 346.0461 (45.2691) |
| θ_ab_ | Natural decay of antibodies after the booster dose | 0.0048 (0.0001) | 0.0066 (0.0002) |
| ɛ_βag_ | Random effects of β_ag_ | 0.6165 (0.0388) | |
| ɛ_αM_ | Random effects of α_M_ | 0.5127 (0.0476) | |
| ɛ_γab_ | Random effects of γ_ab_ | 0.8365 (0.0320) | |
| ɛ_δab_ | Random effects of δ_ab_ | 0.1792 (0.0476) | |
| ɛ_Ab(0)_ | Random effects of Ab(0) | 1.9111 (0.0877) | |
| ɛ_θab_ | Random effects of θ_ab_ | 0.3321 (0.0388) | |


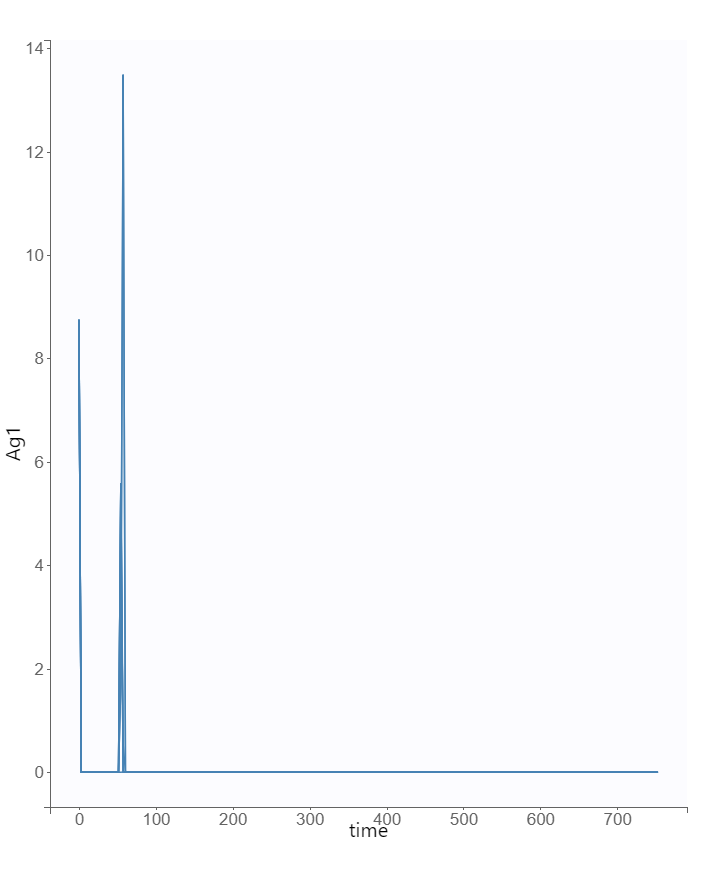

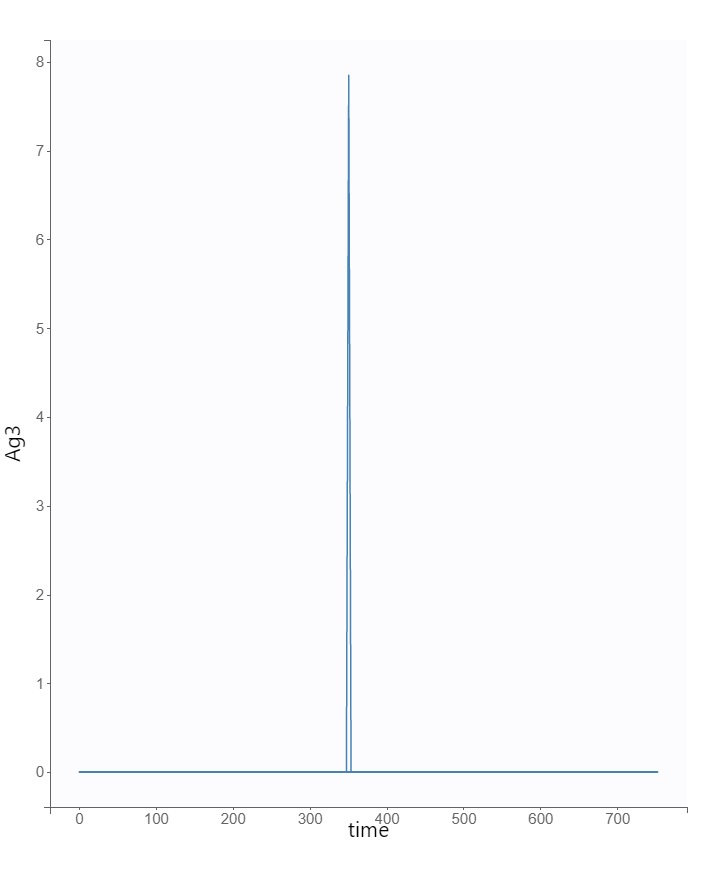


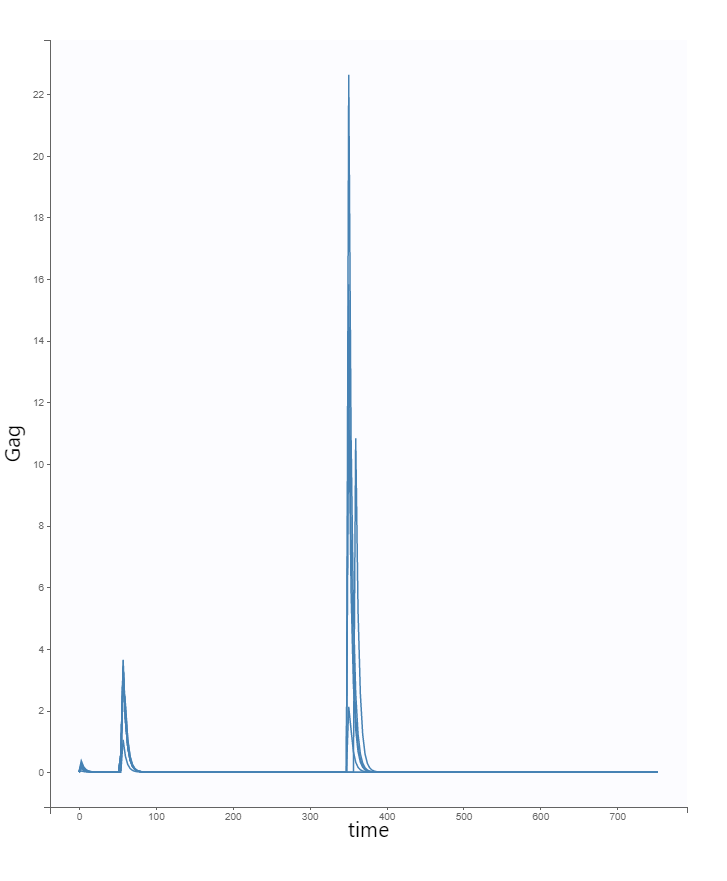

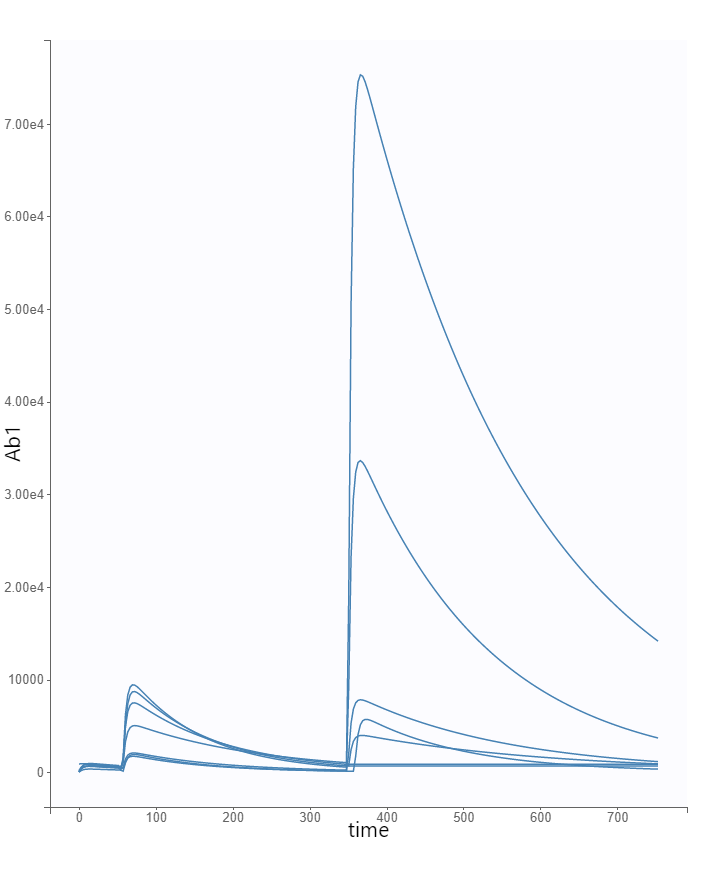


**Figure C1. ODE solutions of the different populations, Ag_1_ and Ag_3_ I the upper panel, and Gag and Ab_1_ in the lower panel. The solutions are associated with a set of individuals.**

**
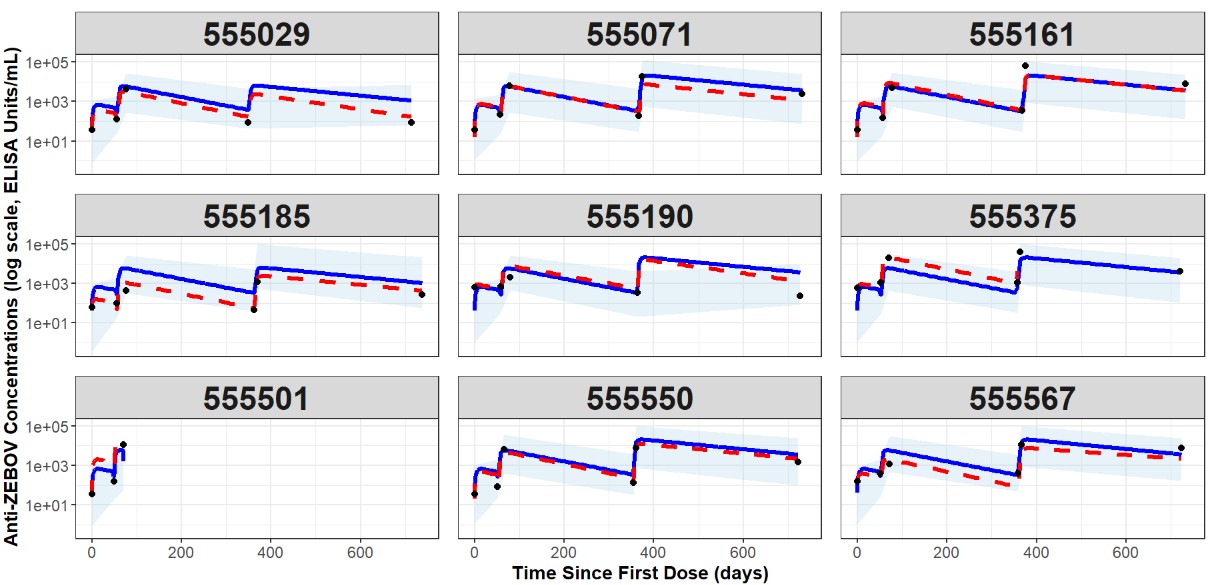
**

**
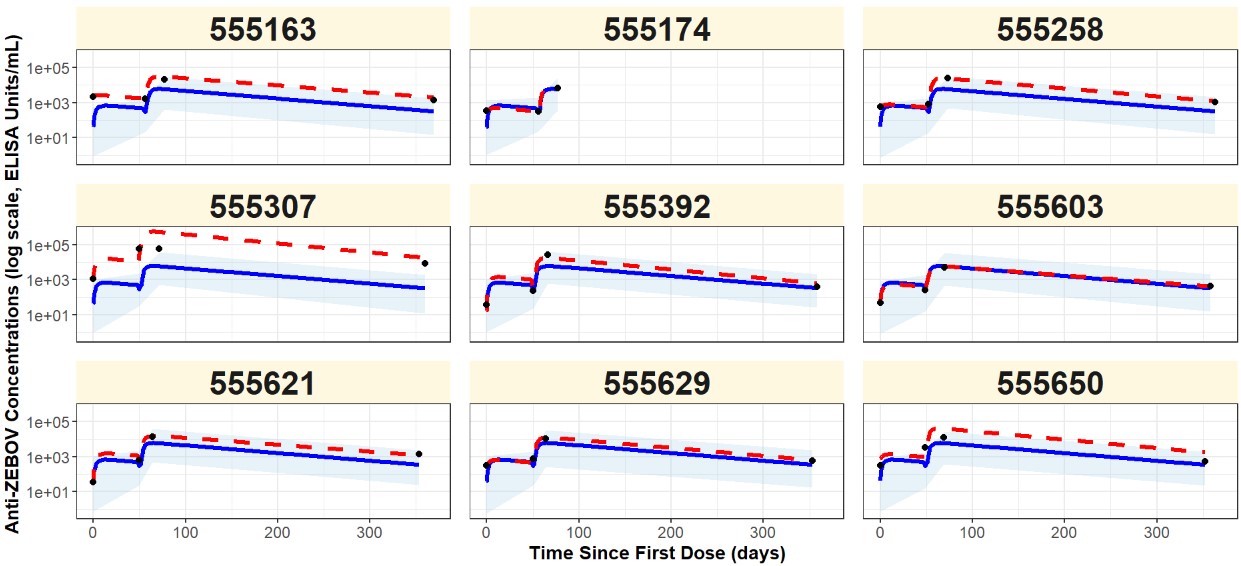
**

**Figure C2. Additional fits of predicted log-transformed IgG titer profiles given by the estimated individual-level model, which is the parsimonious model from the Eqs. (3) with fixed parameters {α = 0.4, δ_ag_ = 0.4, η_ab_ = 3.88, τ_ag_ = 0.24}. In the upper panel cohort 1 is represented in the grey boxes, and in the lower panel the cohort 2 is represented in light yellow boxes. The black dots represent the observed data, the dashed red curves are the approximated conditional mean curves from SAEM, and the blue curves depict the population-level fits based on the estimated fixed effects and individual-specific design characteristics, without incorporating individual-specific random effect estimates. The blue shaded areas represent the 2.5% and 97.5% predicted percentiles, which account for inter-individual variability by including random effects sampled from the estimated population-level distribution, rather than being based on individual-specific estimates.**

***Section D. Model diagnostics, convergence assessment, and sensitivity analyses***

***D.1 Model diagnostics***

In Figure 2, the left panel shows that the predicted distribution aligns closely with the observed data, indicating a good fit between the predictions and the observed values. The right panel depicts observations versus their corresponding predictions, calculated using individual parameters. This plot further suggests that the model specifications are appropriate, as evidenced by the low proportion of outliers and the symmetrical distribution of observations around the predicted values.

***
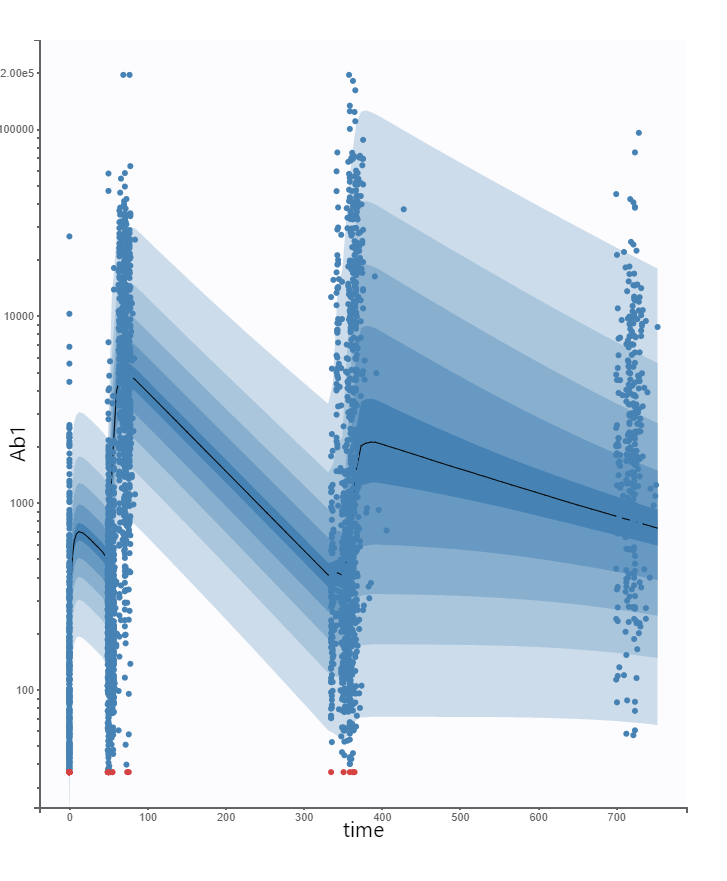

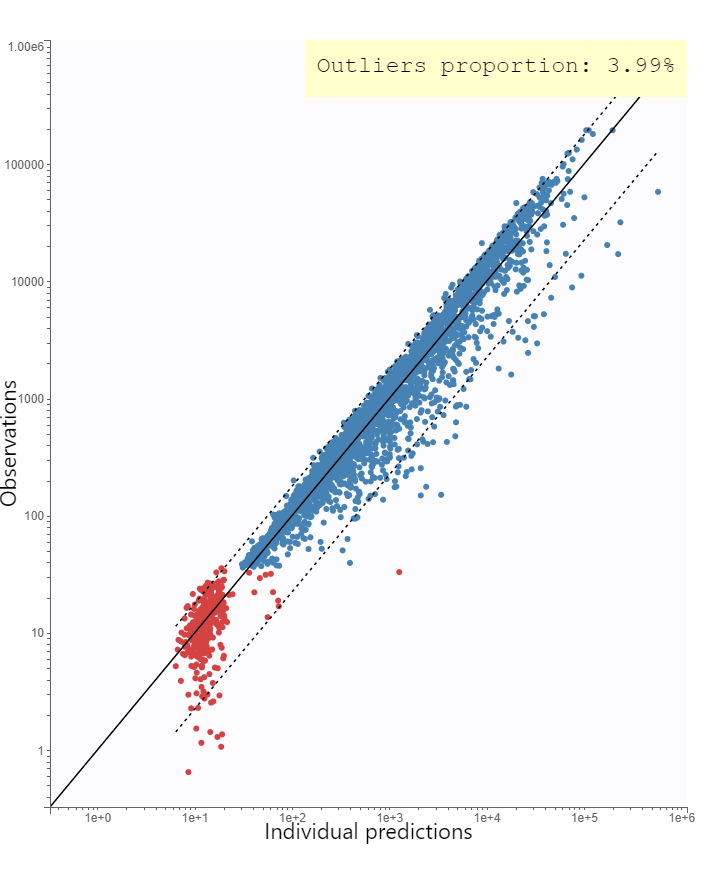
***

**Figure D2: On the left-hand side the predicted distribution is displayed based on multiple simulations of all individuals using the population parameters without considering uncertainty. The blue shaded areas represent the percentiles of the distribution and the blue and red dots represent the observed and censored data respectively. On the right-hand side, the Figure displays the log-observed data versus the corresponding individual predictions from the model in Eqs 3 with fixed parameters: {α = 0.4, δ_ag_ = 0.4, η_ab_ = 3.88, τ_ag_ = 0.24}. The individual parameter estimation are drawn from the conditional distributions, i.e., each observation is associated with a set of individual predictions derived from a set of individual parameters simulated from the same individual conditional distribution. The red dots define the censored observations and the blue dots show the observed data. The dashed lines correspond to the 90% predicted interval, which depends on the residual error model. Predictions that are outside of the interval are denoted as outliers (3.99% in this case).**

***D.2 Convergence assessment***

In this subsection, parameter estimation is evaluated across multiple chains, each initiated with randomly generated initial values for the fixed effects and distinct seeds. This approach aims to assess the robustness of parameter convergence.

Hereunder, the results of the predicted percentiles and model assumptions of the parsimonious model derived from Eqs (3) are shown in the figures below.

**
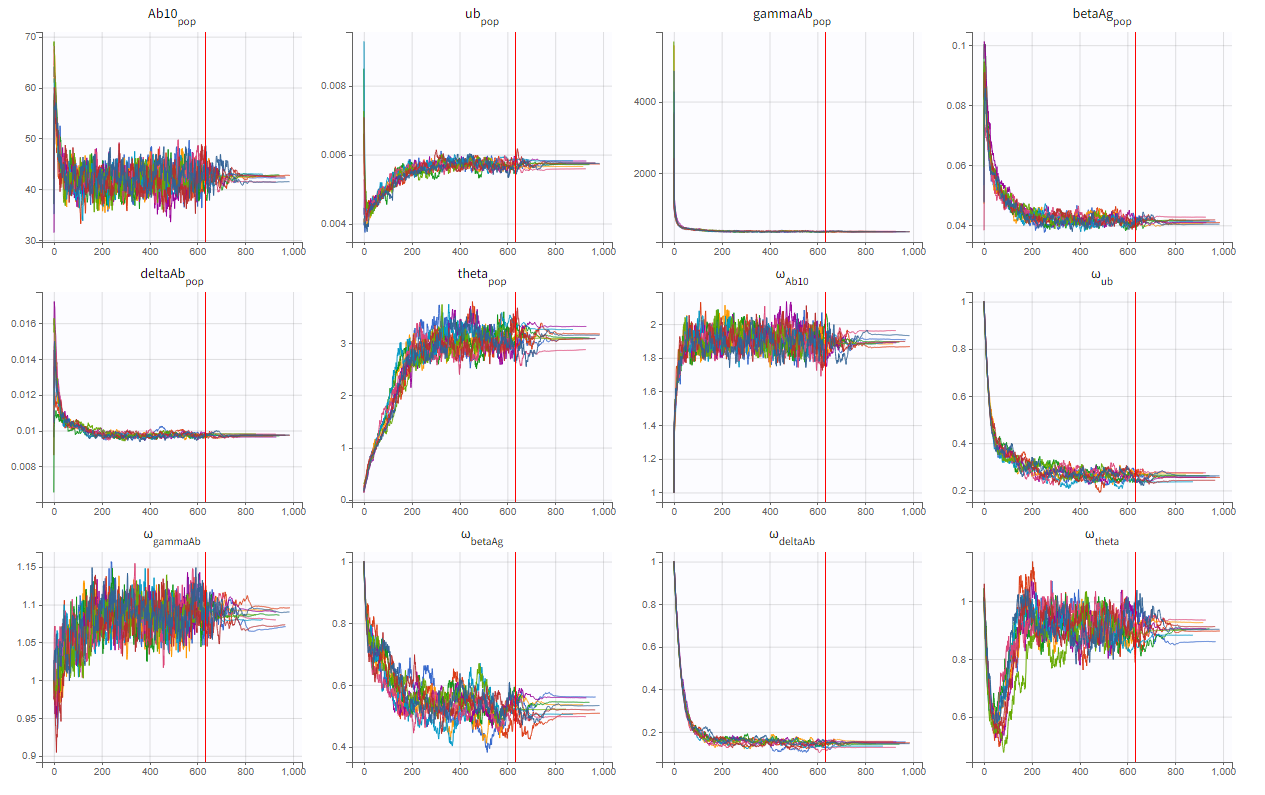
**

**Figure D3. SAEM algorithm from the convergence assessment of the parsimonious model. The different colors represent five different chains (10), for which each of them starts with different randomly generated initial values of fixed effects, which are drawn from intervals defined around the estimated values. The red vertical line indicates at which iteration the SAEM algorithm has reached the maximum likelihood (ML).**

**
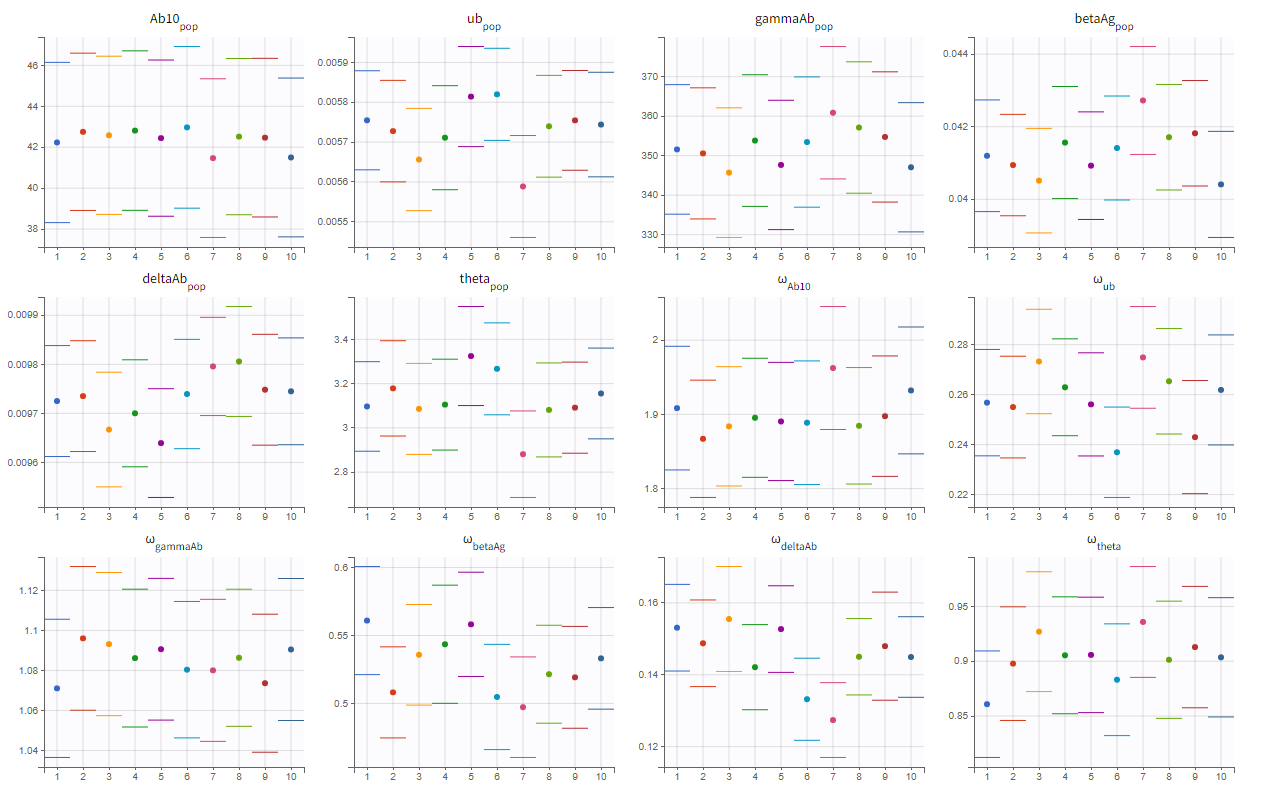
**

**Figure D4. Conditional means from the convergence assessment of the parsimonious model. The different colours represent each of the chain (10). This figure shows in particular the estimated values with the estimation of their respective standard errors displayed in horizontal bars. The estimation of the standard errors are derived from the conditional distribution and use the Fisher information matrix (FIM) coupled with MCMC.**

***D.3 Sensitivity analyses***

The following figures allow for the assessment of potential relationships between population parameters estimated during the SAEM algorithm. This step was crucial in identifying correlations between parameters that could hamper the robustness and convergence of parameter estimates. As described throughout the manuscript, this process was performed iteratively until convergence was achieved.

The figures highlight that some correlations remain evident, such as {γ_ab_, δ_ab_} and {γ_ab_, α_M_}. However, these correlations are not troublesome, as they are consistent with plausible biological relationships between the parameters.


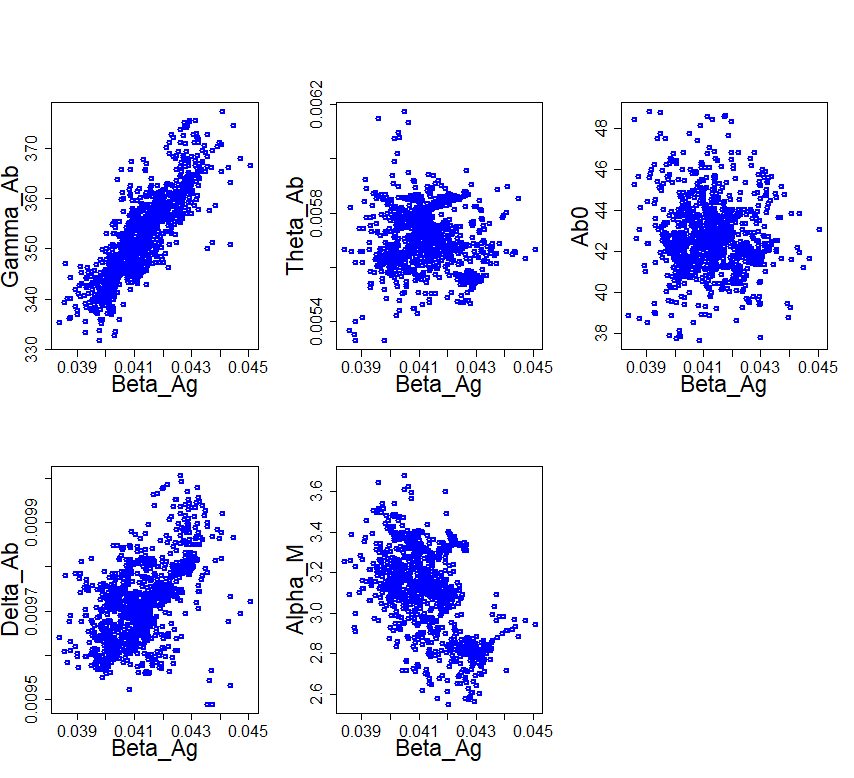

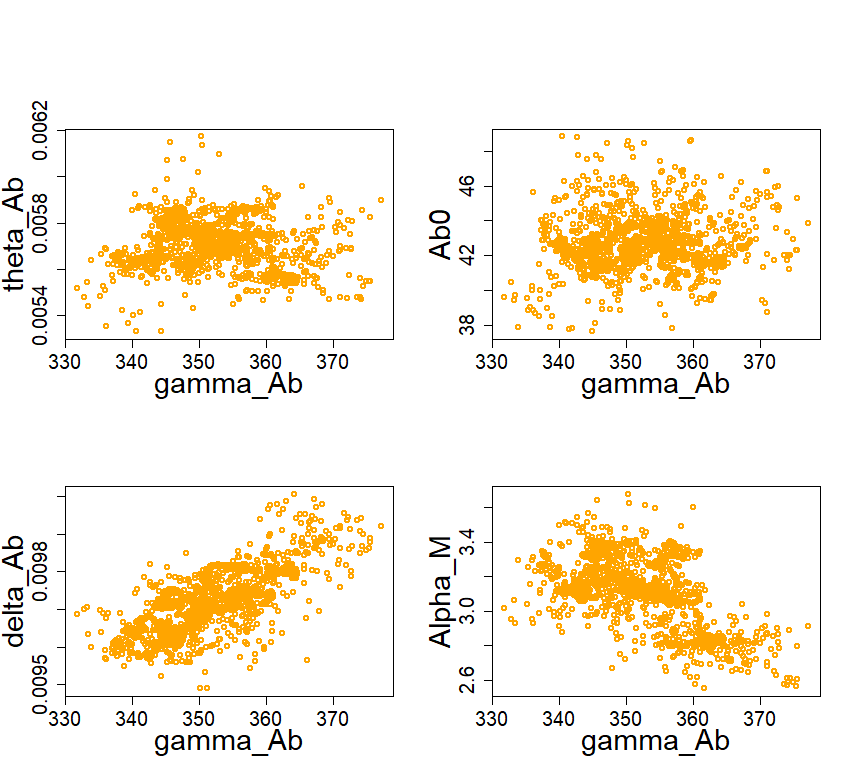


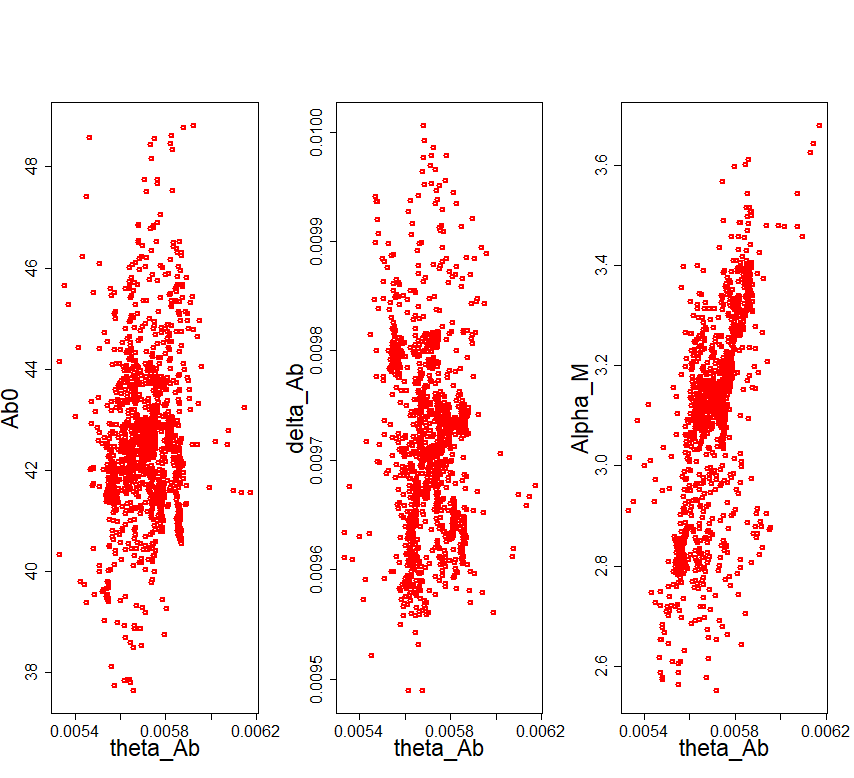

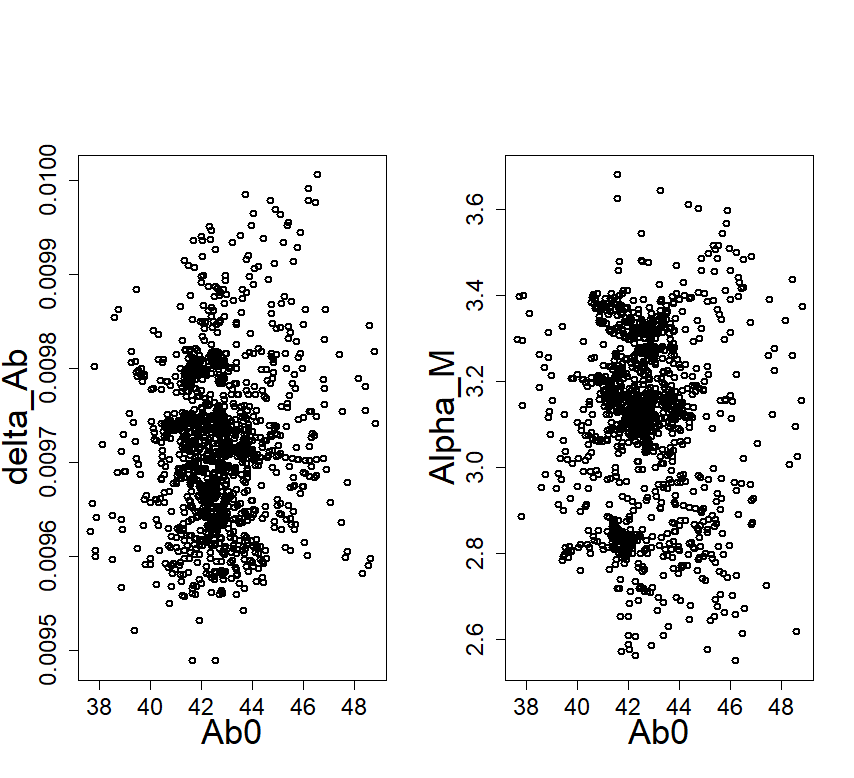


**Figure D5: Scatter plots illustrating the evolution of each combination of population parameters across iterations, starting from iteration 600 when the maximum likelihood is achieved. The upper-left panel displays all combinations involving the parameter β_ag_ (blue), while the upper-right panel shows combinations with γ_ab_ (orange). The red scatter plots represent combinations with the parameter θ_ab_, and the black points correspond to combinations involving Ab(0).**

**Bibliography**

1. Andraud, M., et al., *Living on three time scales: the dynamics of plasma cell and antibody populations illustrated for hepatitis a virus.* PLoS Comput Biol, 2012. **8**(3): p. e1002418.

2. Nguyen, V.K. and E.A. Hernandez-Vargas, *Windows of opportunity for Ebola virus infection treatment and vaccination.* Sci Rep, 2017. **7**(1): p. 8975.

3. Le, D., J.D. Miller, and V.V. Ganusov, *Mathematical modeling provides kinetic details of the human immune response to vaccination.* Front Cell Infect Microbiol, 2014. **4**: p. 177.

4. Farrington, C.P., N.J. Andrews, A.D. Beale, and M.A. Catchpole, *A statistical algorithm for the early detection of outbreaks of infectious disease.* Journal of the Royal Statistical Society Series a-Statistics in Society, 1996. **159**: p. 547-563.
